# Annual Address of the President of the Alumni Association of the American Veterinary College

**Published:** 1897-07

**Authors:** L. H. Howard

**Affiliations:** Boston, Mass.


### THE JOURNAL

OF

### COMPARATIVE MEDICINE AND VETERINARY ARCHIVES.

VOL. XVIII. *JULY*, 1897. No. 7.

### ANNUAL ADDRESS OF THE PRESIDENT OF THE ALUMNI ASSOCIATION OF THE AMERICAN VETERINARY COLLEGE.

BY L. H. HOWARD, D.V.S.,
BOSTON, MASS.

THE assembling to-day of our Association in its twentieth annual reunion is an occasion worthy perhaps of more than passing notice. The celebration of a twentieth anniversary of the organization of a brotherhood such as ours is one full of interest, and marks a point more or less prominent in its history. Let us, therefore, in this twentieth milestone look at the circumstances of our position, mark our progress for twenty years past, and possibly shape our course for another decade.

For twenty years we have been growing from the small beginning of a graduating class few in number, but possessed of commendable enterprise, and to whom occurred this thought—that as our alma mater was already taking rank as one of the representative educational institutions of the country, the large numbers of its prospective graduates should be united, forming an association for the benefit, pleasure, and profit alike of its own members and of the institution to which all would owe their professional being.

Fostered in our early, weak existence by the teaching faculty of the college, and encouraged by its interest in our behalf, strengthened yearly by the addition to our ranks of an increasing number of graduates, and among them many who have shown their ability and readiness to advance the best interests of the association, fortunate in being officered in many instances by men who have demonstrated in the history of veterinary medical associations of this country their exceptional executive qualifications, we have gradually attained to a size, contemplation of which (600 members) inspires the thought, "a powerful factor in the development of veterinary medicine."

Since that small beginning of ours, and during these twenty years which have elapsed, much of importance has occurred in the growth of veterinary science, and each year's record is certainly one of great progress. In some particulars the progress has been exceedingly rapid, and the graduate of twenty years ago, in keeping with the changing conditions, has rapidly advanced, until at the present time he finds himself to be not only the medically and surgically trained veterinarian in demand a decade ago, but, owing to the development in the public mind of an appreciation of the importance of the healthfulness of all animal products, he is in demand as the properly qualified person to inspect such products, the sanitary police officer, the protector of public health.

As a private worker and student in comparative medicine, in the various public official positions, as municipal health officer, as State veterinarian or member of State board having control of contagious diseases of animals, in the ranks of the great Bureau of Animal Industry, in the laboratory, and in the field, the veterinarian has found his scope of usefulness gradually changing and enlarging, and the public demanding qualifications commensurate with its better knowledge of the wonderful evolution of medical science.

The veterinarian has not been found wanting, but is to-day to be found in every portion of our country doing the work expected of him, not only, as I have before said, as the well-trained veterinarian skilled in the treatment of diseased animals, but as the worker in the relatively new science of prophylactic medicine.

Now, then, whether or not in the development of veterinary science in the past twenty years, whether or not in the advance made in comparative medicine purely, whether or not in the resulting growth in the number of institutions fitted for the training of the veterinary practitioner, the teacher of comparative medicine, and the public health official, whether or not in filling all the many demands of the highest development of veterinary and comparative medicine, our alma mater has done its share, we have only to familiarize ourselves with the history of such development in this country—to answer, we can truly say its history is our history. Not only do we include in our ranks many men now well known and of high reputations in the great army of general practitioners, but a glance at the list of veterinarians connected with the educational institutions of the country as organizers, teachers, or directors, shows that many of them received their training within these walls. In the various State veterinary medical associations and in the national organization our men have been prominently active and the workers to whom much of their success has been due. In official positions, municipal, State, and national, many of our fellow alumni are to be found who by their work are establishing a meritorious name for themselves and adding to the credit of their alma mater, besides returning to the people of their respective communities benefits not capable of being measured or calculated in their effect upon the future of the human race.

Therefore, on this our twentieth anniversary let us congratulate ourselves on our present situation and the part we have played in whatever of improvement, progress, or benefit has come to our chosen profession since our organization twenty years ago.

To the eyes of some, today, by the successful application to vehicles of motive power other than horses, the field of usefulness of the practising veterinarian is becoming limited. The bicycle, electric street-car, and horseless vehicles of all descriptions are rapidly being brought to a high state of perfection, and the faster they develop the more threatening becomes the bugbear. To others, however, more optimistic, the prospects of continued employment in the future are as promising as they have ever been. According to the history of the human race in modern times, as civilization has advanced so have multiplied the uses of man's great helper, the horse; and we are not going to do without him now any more than we could when substituting railway cars for stages coaches.

Be that as it may, the field of usefulness for the veterinarian as developing in comparative medicine relating to the protection of human health is one widening every year, and in which there is yet room for many, and no end to the new truths possible to be evolved by able men. Comparative medicine and human medicine are closely related, and this fact is becoming recognized much more fully every year, and in due proportion is the aid of the veterinarian sought.

There is never an end to the wonders which science reveals, and the record of progress in things veterinary is to be continued for years to come. Original investigation, scientific research, experience made valuable to all by its interpretation by bright minds, the higher standard of matriculation, additional courses of study, longer training, and severe requisites for graduation of our educational institutions will all contribute to this end.

As veterinary progress is assured, so is our progress as an association to be continued. To-day we have added to our ranks a goodly number of the newest product of our alma mater. To these young members, "fresh from the halls of learning," we look for new inspiration, and, imbued as they must be with love for alma mater, theirs is the duty to assist in conserving to her the good name and high reputation which has always been hers.

